# Network structure of multivariate time series

**DOI:** 10.1038/srep15508

**Published:** 2015-10-21

**Authors:** Lucas Lacasa, Vincenzo Nicosia, Vito Latora

**Affiliations:** 1 School of Mathematical Sciences, Queen Mary University of London, Mile End Road, E14NS London, UK

## Abstract

Our understanding of a variety of phenomena in physics, biology and economics crucially depends on the analysis of multivariate time series. While a wide range tools and techniques for time series analysis already exist, the increasing availability of massive data structures calls for new approaches for multidimensional signal processing. We present here a non-parametric method to analyse multivariate time series, based on the mapping of a multidimensional time series into a multilayer network, which allows to extract information on a high dimensional dynamical system through the analysis of the structure of the associated multiplex network. The method is simple to implement, general, scalable, does not require *ad hoc* phase space partitioning, and is thus suitable for the analysis of large, heterogeneous and non-stationary time series. We show that simple structural descriptors of the associated multiplex networks allow to extract and quantify nontrivial properties of coupled chaotic maps, including the transition between different dynamical phases and the onset of various types of synchronization. As a concrete example we then study financial time series, showing that a multiplex network analysis can efficiently discriminate crises from periods of financial stability, where standard methods based on time-series symbolization often fail.

Time series analysis is a central topic in physics, as well as a powerful method to characterize data in biology, medicine and economics, and to understand their underlying dynamical origin. In the last decades, the topic has received input from different disciplines such as nonlinear dynamics, statistical physics, computer science or Bayesian statistics and, as a result, new approaches like nonlinear time series analysis^1^ or data mining^2^ have emerged. More recently, the science of complex networks^3^,^4^,^5^ has fostered the growth of a novel approach to time series analysis based on the transformation of a time series into a network according to some specified mapping algorithm, and on the subsequent extraction of information about the time series through the analysis of the derived network. Within this approach, a classical possibility is to interpret the interdependencies between time series (encapsulated for instance in cross-correlation matrices) as the weighted edges of a graph whose nodes label each time series, yielding so called functional networks, that have been used fruitfully and extensively in different fields such as neuroscience^6^ or finance^7^,^8^,^9^. A more recent perspective deals with mapping the particular structure of univariate time series into abstract graphs^10^,^11^,^12^,^13^,^14^,^15^,^16^, with the aims of describing not the correlation between different series, but the overall structure of isolated time series, in purely graph-theoretical terms. Among these latter approaches, the so called visibility algorithms^15^,^16^ have been shown to be simple, computationally efficient and analytically tractable methods^17^,^18^, able to extract nontrivial information about the original signal^19^, classify different dynamical origins^20^ and provide a clean description of low dimensional dynamics^21^,^22^,^23^,^24^. As a consequence, this particular methodology has been used in different domains including earth and planetary sciences^25^,^26^,^27^,^28^, finance^29^ or biomedical fields^30^ (see^31^ for a review). Despite their success, the range of applicability of visibility methods has been so far limited to univariate time series (see however^24^,^28^), whereas the most challenging problems in several areas of nonlinear science concern systems governed by a large number of degrees of freedom, whose evolution is indeed described by multivariate time series.

In order to fill this gap, in this work we introduce a visibility approach to analyze multivariate time series based on the mapping of a multidimensional signal into an appropriately defined multi-layer network^32^,^33^,^34^,^35^,^36^,^37^, which we call *multiplex visibility graph*. Taking advantage of the recent developments in the theory of multilayer networks^32^,^34^,^35^,^36^,^37^,^38^,^39^, new information can be extracted from the original multivariate time series, with the aims of describing signals in graph-theoretical terms or to construct novel feature vectors to feed automatic classifiers in a simple, accurate and computationally efficient way. We will show that, among other possibilities, a particular projection of this multilayer network produces a (single-layer) network similar in spirit to functional networks, while being more accurate than standard methodologies to construct those ones. We validate our method by investigating the rich high-dimensional dynamics displayed by canonical models of spatio-temporal chaos, and then apply our framework to describe and quantify periods of financial instability from a set of empirical multivariate financial time series.

## Results

Let us start by recalling that visibility algorithms are a family of geometric criteria which define different ways of mapping an ordered series, for instance a temporal series of *N* real-valued data 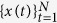, into a graph of *N* nodes. The standard linking criteria are the natural visibility^15^ (a convexity criterion) and the horizontal visibility^16^ (an ordering criterion). In the latter version, two nodes *i* and *j* are linked by an edge if the associated data *x*(*i*) and *x*(*j*) have horizontal visibility, i.e. if every intermediate datum *x*(*k*) satisfies the ordering relation *x*(*k*) < inf{*x*(*i*), *x*(*j*)}, 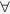 *k*: *i* < *k* < *j*. The resulting Horizontal Visibility Graph (HVG) is an outerplanar graph (indeed a subgraph of the original visibility graph), whose topological properties have been shown to be analytically tractable for a large class of different dynamical processes^16^,^17^. Both the natural and horizontal graphs are undirected, however directed graphs can be easily constructed by distinguishing ingoing from outgoing links with respect to the arrow of time, something which has proven useful to assess time asymmetries and to quantify time series irreversibility^40^,^41^,^42^.

**Figure 1:**
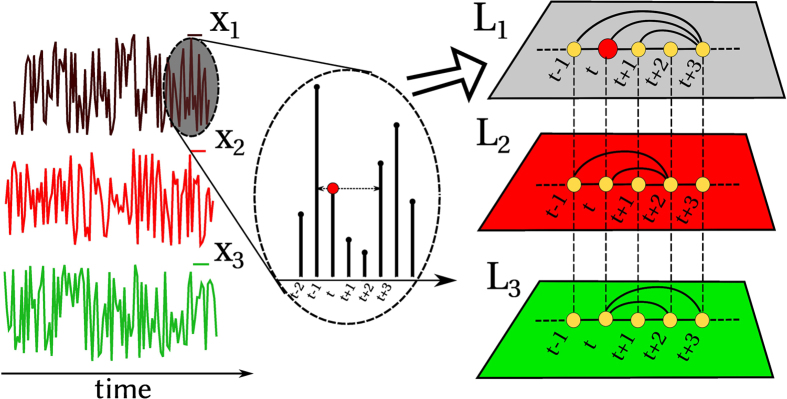
The Horizontal Visibility Graph (HVG) algorithm maps a *M*-dimensional time series 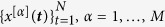, into a *multiplex visibility graph* 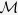, i.e. a multi-layer network where each layer *α* is the HVG of the *α*-th component of the time series.

Consider a *M*-dimensional real valued time series 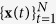, with 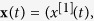 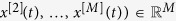 for any value of *t*, measured empirically or extracted from a *M*-dimensional, either deterministic or stochastic, dynamical system. An *M*-layer multiplex network, that we call the *multiplex visibility graph* 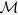 is then constructed, where layer *α* corresponds to the HVG associated to the time series of state variable 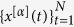. We illustrate this procedure for *M* = 3 in Fig. 1. Note that in this work we focus on the undirected, horizontal visibility, but other visibility linking criteria can be analogously used to define different multiplex graphs. 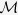 is represented by the vector of adjacency matrices of its layers 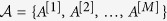, where 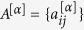 and 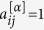 if and only if node *i* and node *j* are connected by a link at layer *α*^33^,^37^. Such a mapping builds a bridge between multivariate series analysis and the recent developments in the theory of multilayer networks^32^,^34^,^35^,^36^,^37^,^38^,^39^, making it possible to employ the structural descriptors introduced to study multiplex networks as a toolbox for the characterisation of multivariate signals. In the following we will focus on two measures which capture, respectively, the abundance of single edges across layers and the presence of inter-layer correlations of node degrees^39^. In such a way we can characterize information shared across variables (layers) of the underlying high dimensional system, this aspect being indeed of capital importance in fields such as neuroscience or economics and finance.

The first measure we introduce is the average edge overlap


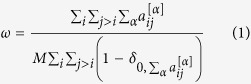


which computes the expected number of layers of the multiplex on which an edge is present. Note that *ω* takes values in [1/*M*, 1], and in particular *ω* = 1/*M* if each edge (*i, j*) exists in exactly one layer, i.e. if there exist a layer *α* such that 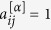 and 
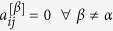
, while *ω* = 1 only if all the *M* layers are identical. As a consequence, the average edge overlap of a multiplex visibility graph can be used as a proxy of the overall coherence of the original multivariate timeseries, with higher values of *ω* indicating high correlation in the microscopic structure of the signal.

The second measure we use quantifies the presence of interlayer correlations between the degrees of the same node at two different layers. Given a pair of layers *α* and *β* of 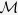, respectively characterized by the degree distributions *P*(*k*^[*α*]^) and *P*(*k*^[*β*]^), we can define an *interlayer mutual information I*_*α*,*β*_ as:


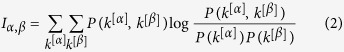


where *P*(*k*^[*α*]^, *k*^[*β*]^) is the joint probability to find a node having degree *k*^[*α*]^ at layer *α* and degree *k*^[*β*]^ at layer *β* (see Methods for details). In general, the higher *I*_*α*,*β*_ the more correlated the degree distributions of the two layers and, therefore, the structure of the associated time series. If we then average the quantity *I*_*α*,*β*_ over every possible pair of layers of 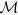, we obtain a scalar variable *I* = 〈*I*_*α*,*β*_〉_*α*,*β*_ which captures the typical amount of information flow in the system.

It is worth noticing that the values {*I*_*α*,*β*_} can be considered as the weights of the edges of a *graph of layers* 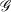, this being a projection of the original multiplex visibility graph 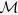 into a (single-layer) weighted graph of *M* nodes, where each node represents one layer. The weights of the edges of such graph denote the magnitude of mutual information, but our approach can be easily generalized so that edges can represent different types of interdependence, such as correlation^6^, causality^43^, etc.^44^ between layers. By using this particular projection, where each layer is represented by a node and relationships between layers are indicated by weighted edges, we are actually analyzing the visibility analog of classical functional networks. However, we shall indeed see that the projection obtained through the multiplex visibility algorithm is genuinely different and often works better than other ways to construct functional networks. This is mainly because the latter ones often require the symbolization of each time series, and this pre-processing is usually afflicted by several limitations and ambiguities^1^. As we show in the online Supplementary Information (SI), the technique proposed here also appears to be free from these well known ambiguities.

### Information flow and phase diagram in Coupled Map Lattices

As a case study we first consider diffusively coupled map lattices (CMLs), high-dimensional dynamical systems with discrete time and continuous state variables, widely used to model complex spatio-temporal dynamics^45^ in as disparate contexts as turbulence^46^,^47^, financial markets^48^, biological systems^49^ or quantum field theories^50^. Namely we consider a ring of *M* sites, and we assume that the dynamical evolution of the state *x*^[*α*]^ of each site *α* is determined by a compromise between an internal chaotic evolution and an external diffusive coupling among nearest-neighbor sites:


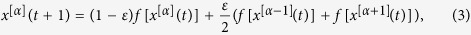



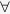
*α* = 1,…, *M*, where *ε* ∈ [0, 1] is the coupling strength, and *f*(*x*) is typically a chaotic map. In the right hand of the equation one can easily recognize the discrete version of the Laplacian (that drives diffusion). The control parameter *ε* is a quantity that tunes the relative strength of the diffusive term in the evolution of the system, acting as an effective viscosity constant, and separating different dynamical phases where the system gets trapped into different attractors as *ε* varies. It is well known^45^ that for different values of *ε* and *M*, CMLs display a very rich phase diagram, which includes different degrees of synchronization and dynamical phases such as Fully Developed Turbulence (FDT, a phase with incoherent spatiotemporal chaos and high dimensional attractor), Pattern Selection (PS, a sharp suppression of chaos in favor of a randomly selected periodic attractor), or different forms of spatio-temporal intermittency (STI, a chaotic phase with low dimensional attractor, which is indeed a pseudo-phase that interpolates between FDT and PS). The origin of such a rich and intertwined structure comes from the interplay between the local tendency towards inhomogeneity, induced by the chaotic dynamics of each variable, and the global tendency towards homogeneity in space, induced by the diffusive coupling.

**Figure 2:**
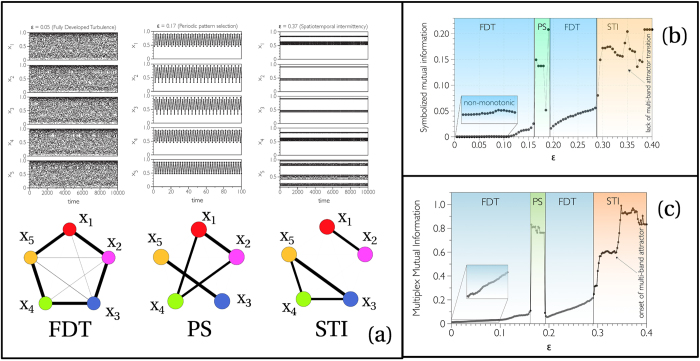
(**a**) Sample time series generated by five diffusively coupled fully chaotic logistic maps at different values of the coupling strength *ε*, showing respectively Fully Developed Turbulence (FDT) at *ε* = 0.05, Pattern Selection (PS) at *ε* = 0.17, and Spatio-temporal Intermittency (STI) at *ε* = 0.37. Information flow among units is well captured by projecting the multiplex visibility graph 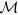 into a layer graph 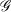 (bottom), whose nodes represent layers and the edges weights (their thickness in the figure) the mutual information among them. (**b**) The average pairwise mutual information *I*^SYMB^ computed from pre-symbolized time series, or (**c**) the corresponding version *I* computed from the associated multiplex visibility graph can both be used as order parameters to distinguish different dynamical phases (see SI for additional analysis with average edge overlap). However, only the multiplex measure is capable of detecting fine-grain structures such as the onset of multi-band chaotic attractors.

Figure 2 summarizes the analysis and results obtained for a CML of *M* = 5 diffusively coupled, fully chaotic logistic maps *f*(*x*) = 4*x*(1 − *x*), which exhibits several transitions from high dimensional chaos, to pattern selection, to several forms of partially synchronized chaotic states when *ε* is increased. Sample multivariate time series of the system in three different states (Fully Developed Turbulence (FDT) at *ε* = 0.05, Periodic Pattern Selection (PS) at *ε* = 0.17, and Spatio-temporal Intermittency (STI) at *ε* = 0.37) are shown in the top of panel (a). In Fig. 2(c) we plot, as a function of *ε*, the average mutual information *I* of the multiplex visibility graph associated to a multivariate time series of 2^14^ data extracted from eq. (2) (results are averaged over 100 realizations, see Methods for details and SI equivalent analysis using average edge overlap). The different dynamical phases that emerge as a function of *ε* are also depicted as different background colors. We find that *I* is able to distinguish between different dynamical phases. In particular, *I* is a monotonically increasing function of *ε* in the FDT phases, and therefore quantifies the amount of information flow among units, which increases in the FDT as the contribution of the diffusion increases. Notably, it also detects qualitative changes in the underlying dynamics (such as the chaos suppression in favor of a randomly selected periodic pattern, or the onset of a multi-band chaotic attractor during intermittency) and therefore constitutes a scalar order parameter of the system.

For comparison, in Fig. 2(b) we also plot the corresponding quantity *I*^SYMB^, derived from a standard functional network analysis. Namely, *I*^SYMB^ is the average mutual information computed directly on the multivariate time series, after performing the necessary time series symbolization (see methods for details). Although there are qualitative similarities with *I*, subtle aspects such as the monotonic increase of synchronization with *ε* in FDT, or the onset of multiband attractors in STI are not captured by *I*^SYMB^ (additional details comparing our method to standard functional network approaches can be found in the SI). In the bottom of panel (a) of the same figure we also report the projections of the three multiplex networks 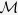 into the corresponding graphs of layers 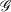, whose edge widths are proportional to the values *I*_*α*,*β*_ of mutual information between layers *α* and *β*. A simple visual inspection of such graphs reveals the different type of information flow among units, depending on the dynamical phases of the system. In particular, notice that the diffusive nature of the coupling emerges in the ring-like structure of graph 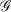 corresponding to weakly interacting maps (FDT) (the analysis is extended in SI to globally coupled maps, these being a mean-field version of CML where complete synchronisation is possible, showing that our method correctly detects the onset of this new regime).

### Scaling up the system.

**Figure 3:**
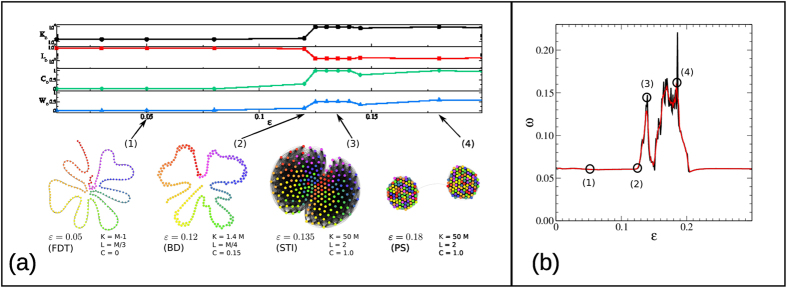
(**a**) Backbone graphs 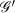 of the graph of layers 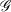 obtained from the multiplex visibility graph of *M* = 200 diffusively coupled chaotic maps for different values of *ε*. For each dynamical phase, 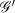 has a different structure. In FDT, 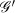 is a chain, revealing the diffusive nature of the coupling in this weakly interacting situation. In PS, 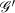 is formed by non-interacting communities, whereas in STI -where the ghost of the periodic attractor is perturbed by chaotic excursions-, 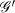 is formed of slightly overlapping dense communities. We also report several topological properties of 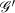 (number of edges *K*, average shortest path length *L*, clustering coefficient *C*, and total weight of the edges) that have different qualitative values in each phase. Nodes colours determine the map label, so similar colours denote maps at close distance in the CML. (**b**) The four different phases are also visible in the plot of the average edge overlap of the multiplex as a function of the coupling strength *ε* (numerical simulations are averaged over 50 realizations). In particular, for *ε* < 0.12 we have *ω* at its minimum, as expected in FDT. Then, for 0.12 < *ε* < 0.15 we observe a consistent increase in *ω*, which is in agreement with the development of BD first and STI afterwards. For 0.16 < *ε* < 0.2 the system is in the periodic pattern selection phase, characterised by relatively larger values of edge overlap, while for *ε* > 0.2 the systems is again in FDT. The values of *ε* corresponding to the four sample graphs shown in panel (**a**) are indicated by empty circles.

The previous study suggests that the quantities {*I*_*α*,*β*_} (see Fig. 2(a)) accurately capture relevant information of the underlying dynamics. To further explore this aspect and to assess scalability, we considered a chain of *M* = 200 diffusively coupled logistic maps, each governed by Eq. (3). New short dynamical phases, such as the so called Brownian motion of Defects (BD) –a transient phase between FDT and PS–, emerge when the dimension of the system is increased, whereas others such as intermittency are now just mere interfaces advancing the onset of periodicity (PS). These phases have been extensively analysed in ref. 45. As the description gets more cumbersome, projections and coarse-grained variables are needed. Since the graph of layers 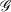 is by construction a complete graph (just as any functional network), for visual reasons in Fig. 3(a) we report the structural properties of 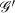, the backbone of 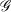 obtained starting from an empty graph of *M* nodes and adding edges sequentially in decreasing order of *I*_*α*,*β*_, until the resulting graph consists of a single connected component. The structure of 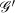 is unique for each phase and qualitatively different across phases, thus providing a simple qualitative way to portrait different dynamics in high-dimensional systems.

We might complement this analysis by addressing now the average edge overlap *ω*, which is a somehow more basic structural indicator. This measure effectively plays the role of an order parameter able to distinguish these different dynamical phases quite accurately, as shown by the plot reported in Fig. 3(b). As expected, the FDT phases, observed for *ε* < 0.12 and for *ε* > 0.2 (see ref. 45) are associated to the minimum values of *ω* 
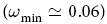
. Conversely, the periodic PS phase (0.16 < *ε* < 0.20) is characterised by the largest values of edge overlap. In this phase, each edge is present, on average, in up to 45 of the 200 layers of the multiplex, indicating a higher overall level of coherence. It is interesting to stress that the average edge overlap of a multiplex network can be computed quite efficiently from the adjacency matrices of the layers. In fact, it is easy to show that the time complexity of computing *ω* is 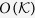, where 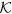 is the total number of edges in the multiplex. As horizontal visibility graphs tend to be sparse for a large variety of dynamical systems^16^, the effective time complexity is *O*(*N* × *M*), i.e. linear in the length and in the dimension of the original time series. If we take into account the fact that the time complexity of the horizontal visibility graph algorithm is linear for noisy series in the length of the series, then we can conclude that the proposed approach to multivariate time series analysis can be readily employed also for the analysis large data sets.

### Multiplex analysis of financial instabilities

As an example of the possible applications of the multiplex visibility graph approach to the analysis of real-world multivariate time series, we report a study of the prices of financial assets. Namely, we considered the time evolution of stock prices of *M* = 35 of the largest US companies by market capitalization from NYSE and NASDAQ (see SI for details) over the period 1998–2013. The *M* time series have a very high resolution (one data per minute), yielding *O*(2 ⋅ 10^6^) data per company. We divided each multivariate time series into non-overlapping periods of three months (quarters), and we constructed a *temporal* multiplex visibility graph consisting of 64 multilayer snapshots, each formed by the 35-layer multiplex visibility graph corresponding to one of the 3-months periods. We then investigated the time evolution of the multiplex mutual information among layers, and how this correlates with the presence of periods of financial instability.

**Figure 4:**
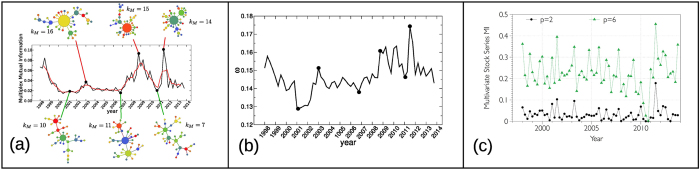
(**a**) The multiplex mutual information is a suitable quantity to detect global changes of behavior in multivariate financial time series. In the plot we report the value of the average information (the red line is the corresponding running average) computed from the multiplex networks constructed from price time series of 35 major assets in NYSE and NASDAQ in each 3-month period between January 1998 and December 2013 (see Methods for details). Notice that the most pronounced peaks of mutual information correspond to periods of major financial instability (the .com bubble in 1998–1999, the mortgage subprime crisis in 2007–2012). The Maximum Spanning Trees of the corresponding networks of layers (six typical examples are shown), always include a large hub during crises (the three top networks), whose degree *k*_*M*_ is larger than the maximum degree observed in periods of stability (the three bottom networks). Each asset is assigned the same color in all the networks, while the size of a node is proportional to its degree. (**b**) The difference between periods of stability and crises is also detected by the average edge overlap *ω*. For visual reference, we report in the plot the six example points examined in panel (**a**). It is evident that peaks of *ω* correspond to crises while dips are usually associated to normal market activity. (**c**) The mutual information among the same set of time series performed after a standard symbolization with *p* symbols is not able to single out crises. The resulting signal is much more herratic and not as informative as the multiplex mutual information shown in panel (**a**).

In Fig. 4(a) we plot the value of the average multiplex mutual information *I* as a function of time, while in Fig. 4(b) we show the values of average edge overlap *ω*. For comparison we also report in Fig. 4(c) the values of *I*^*SYMB*^ computed directly on the original series, after an appropriate symbolization (see Methods and SI for details). We find that the multiplex visibility graph approach captures the onset of the major periods of financial instability (1998–1999, corresponding to the .com bubble, and 2007–2012, corresponding to the great recession that took place as a consequence of the mortgage subprime crisis), which are characterised by a relatively increased synchronisation of stock prices, clearly distinguishing them from the seemingly unsynchronised interval 2001–2007, which in turn corresponds to a more stable period of the economy. In direct analogy with the language used for CMLs, we could say that in periods of financial stability, the system is close to equilibrium, degrees of freedom are evolving in a quasi-independent way, reaching a fully developed turbulent state of low mutual information (hence unpredictable and efficient from a financial viewpoint). On the other hand, during periods of financial instability (bubbles and crisis) the system is externally perturbed, hence driven away from equilibrium, and the degrees of freedom share larger mutual information (the system is therefore less unpredictable and inefficient from a financial viewpoint). Notice that this picture is confirmed by the plot of the average edge overlap *ω*, reported in Fig. 4(b), where peaks correspond quite closely to the major periods of financial crises. As shown in Fig. 4(c), instead, an analogous analysis based on the symbolization of the time series fails to capture all such details (see SI for additional analysis). Finally, as also seen in the case of the multiplex visibility graphs associated to CMLs, the differences in the values of average mutual information corresponding to different phases are indeed related to a different underlying structure of the network of layers. In Fig. 4(a) we show the Maximal Spanning Trees (MST) of the networks of layers associated to six typical time windows^7^,^51^. The three networks at the bottom of the Figure represent periods of financial stability, while those at the top of the Figure correspond to the three local maxima of mutual information. Interestingly, the MSTs in periods of financial instability all have a massive hub which is directly linked to as much as 50% of all the other nodes. Conversely, the degree is more evenly distributed in the MSTs associated to periods of economic stability.

## Discussion

The approach based on multiplex visibility graphs introduced in this work provides an alternative and powerful method to analyze multivariate time series. We have first validated our method focusing on signals whose underlying dynamics is well known and showing that measures describing the structure of the corresponding multiplex networks (which are not affected by the usual problems of standard symbolization procedures) are able to capture and quantify the onset of dynamical phases in high-dimensional coupled chaotic maps, as well as the increase or decrease of mutual information among layers (maps) within each phase. We then have studied an application to the analysis of multivariate financial series, showing that multiplex measures, differently from other standard methods, can easily distinguish periods of financial stability from crises, and can thus be used effectively as a support tool in decision making.

The proposed method is extremely flexible and can be used in all situations where the dynamics is poorly understood or unknown, with potential applications ranging from fluid dynamics to neuroscience or social sciences. In particular, the accuracy of some basic multiplex metrics (e.g., the average edge overlap and the normalised mutual information of the degree sequences) in distinguishing different dynamical phases makes it possible to efficiently analyse large multivariate timeseries. In fact, the construction of the horizontal visibility multiplex network associated to a multivariate time-series is linear in the number of nodes and in the number of variables, while the estimation of average edge overlap and mutual information are respectively linear and quadratic in the number of layers. In this article we have focused only on a particular aspect, which is the characterization of the information flow among the different variables of the system, and we have consequently based our analysis on the study of the resulting networks of layers. However, our approach is quite general, and the mapping of multivariate time series into multiplex visibility graphs paves the way to the study of the relationship between specific structural descriptors recently introduced in the context of multiplex networks and the properties of real-world dynamical systems. We are confident that our method is only the first step towards the construction of feature-based automatic tools to classify dynamical systems of any kind.

## Methods

### Inter-layer degree correlations in multiplex networks

In a *M*-layer multiplex network 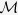 on *N* nodes, defined by the vector of adjacency matrices 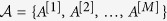, each node *i* is associated to a multiplex degree vector 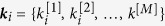, where 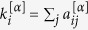 is the total number of edges incident on node *i* at layer *α*. With the usual notation, *P*(*k*^[*α*]^) is the degree distribution of layer *α*. Several measures have been recently proposed in the literature to quantify the sign and intensity of inter-layer degree correlations. Here we choose to characterize the degree correlation between two layers *α* and *β* by using the mutual information between the corresponding degree sequences 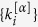 and 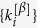, *i* = 1,…, *N*. This choice was motivated by the direct dynamical interpretation of the mutual information for visibility graphs. In particular, we computed the quantities:


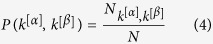


where 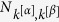 is the number of nodes having degree equal to *k*^[*α*]^ and *k*^[*β*]^ respectively on layer *α* and on layer *β*. By definition we have:


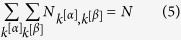


Using this notation, the mutual information *I*_*α*,*β*_ between the degree distributions at layers *α* and *β* reads:


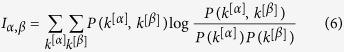


### Comparing with measures on symbolized series

Symbolization is one of the standard requisites for the analysis of (multivariate) time series. The procedure consists in transforming the original (continuous state) signal 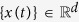 into a (discrete state) sequence {*S*(*t*)} whose values belong to a finite set of *p* symbols {*s*_1_, *s*_2_,…, *s*_*p*_}. There are several ways to construct such symbolised representations of multivariate signals, but one of the main problems which characterise symbolisation is the choice of the mapping function and the determination of the appropriate number of symbols to be used (see SI for details). The standard approach in CMLs (for which {*x*(*t*)} ∈ [0, 1]^*d*^) is to make a partition of each dimension into *p* non-overlapping subintervals, assigning a different symbol to those values belonging to different subintervals (see SI for additional details). The resulting symbolized series is a symbol sequence. One can then estimate the mutual information between two symbol sequences *x*(*t*) and *y*(*t*) as


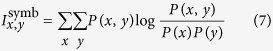


so that *I*^symb^ is just the pairwise average of 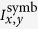 over a set of series. In Fig. 2(b) we reported the value of the averaged mutual information *I*^*SYMB*^(*ε*) for a system of 5 diffusively coupled chaotic map lattices. In particular, for each value of *ε*, we generated 100 realizations of a multivariate series 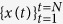 of the dynamics described in Eq. (3), with *N* = 2^14^ points and *x* ∈ [0, 1]^5^. The time series corresponding to each realization 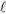 was symbolized by partitioning the phase space of each variable into *p* = 2 non-overlapping subintervals of the same size 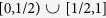. Accordingly, an original multivariate time series is coarse-grained into a multivariate symbolic sequence. Then, we averaged the mutual information 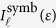 for each realization, as explained in the previous subsection, and finally 
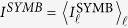
. A similar computation is made in Fig. 4(b) for financial time series, where we have analyzed the cases with *p* = 2 and 6 symbols.

For the construction of Fig. 2(c) we used, for each value of *ε*, the same 100 realizations of Eq. (3). For each realization 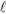, we constructed the corresponding multiplex visibility graph, by considering as layers the Horizontal Visibility Graphs of each of the five components. Then, we computed the pairwise mutual information between any pair of layers 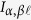, and the corresponding average 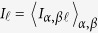. The value of *I* reported in the figure is the average over the 100 realizations, i.e. 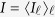.

A similar computation is made in Fig. 4(a) in the case of financial time series.

## Additional Information

**How to cite this article**: Lacasa, L. *et al.* Network structure of multivariate time series. *Sci. Rep.* **5**, 15508; doi: 10.1038/srep15508 (2015).

## Supplementary Material

Supplementary Information
