# Supplementary material for: Network structure of multivariate time series
Source: Sci Rep. 2015 Oct 21;5:15508. doi: 10.1038/srep15508 (PMC4614448; doi:10.1038/srep15508)
Supplement: Supplementary Information [file srep15508-s1.pdf]

Supplementary Information to
"Network structure of multivariate time series"

Lucas Lacasa, Vincenzo Nicosia, and Vito Latora
School of Mathematical Sciences, Queen Mary University of London, Mile End Road, E14NS London, UK

In this supplementary material we first compare the performance of the visibility multiplex network method with results obtained according to standard symbolization approaches. We then focus on diffusively coupled chaotic maps and assess the performance of both methods (the currently proposed visibility multiplex approach, and the standard approach based on series symbolization) in the task of retrieving and accurately describing several well-known dynamical properties of these systems, namely (i) the transition between different dynamical phases, (ii) the increase of synchronisation as a function of the coupling constant, and (iii) the accurate location of the onset of multiband chaotic attractors. Whereas the visibility approach correctly captures numerically all these properties, the approach based on symbolisation is only successful for a narrow set of the symbolisation parameters (number of symbols, phase space partition). We conclude that, within this study, the visibility multiplex approach is at least as accurate as the *optimal* symbolisation criterion, although the former is parameter free and therefore is more efficient in practice. In a second part, we explore the performance of alternative graph-theoretical measures, such as the average edge overlap, as scalar order parameters. In a third part, we explore how the proposed methodology is able to detect the onset of full synchronisation in globally coupled chaotic maps (GCM), these being a mean-field version of diffusively coupled maps where complete synchronisation is possible. Finally, we provide additional details on the multiplex analysis of multivariate financial series. In particular, we provide a detailed analysis on how a direct analysis of mutual information in a symbolised time series fails to capture differences and classify financial years in terms of their stability.

PACS numbers: 89.75.Hc, 05.45.Tp, 89.75.Fb, 05.45.Ra

I. SYMBOLISATION: STANDARD PRACTICE, POSSIBLE PITFALLS

The standard approach to numerically study coupled map lattices (CMLs) (and more generally, trajectories of any dynamical system, irrespective of its dimension) is to preprocess the multivariate time series generated by an orbit in $\mathbb{R}^d$ through a so called *symbolization*

$$\{\mathbf{x}(t)\} \longmapsto \{\mathcal{S}(t)\},$$

where $\mathbf{x} \in \mathbb{R}^d$ and $\mathcal{S} \in \{s_1, s_2, ..., s_p\}^d$. Once the original (continuous state) series $\{\mathbf{x}(t)\}$ is symbolized into a (discrete state) sequence of symbols $\{\mathcal{S}(t)\}$, several measures such as the ones involving frequency histograms of finite size samples (as unbiased estimators of probability densities) such as complexity or information [1, 5] measures can be computed and used to analyse the system. A large majority of the methods that analyse empirical time series require such preprocessing, although it is fair to say that this is not always clarified or explicitly stated.
However, such preprocessing is far from trivial, in the sense that results usually depend on such *ad hoc* procedure. First, how many symbols p should we define? Note that standard option, widely used in the field of symbolic dynamics, is to makes use of two symbols ($p = 2$), where $s_1 \equiv L$ and $s_2 \equiv R$ (alternatively and without loss of generality, $s_1 \equiv 1$ and $s_2 \equiv 2$). In general, each symbol indeed corresponds to the label of a different, non-overlapping cell c_i, such that the set $\mathcal{C} = \cup_{i=1}^{p} c_i$ is a partition or tile of the phase space under study. For instance, for $p = 2$ the function $f : [a, b] \to [a, b]$ is usually symbolized according to a homogeneous partition: $c_1 = [a, b/2), c_2 = [b/2, b]$. Now, again, is this is best choice for the definition of cells? The response is also not unique, and as a matter of fact, each dynamical system will typically require a different symbolisation and partition. For instance, suppose that the distribution of phase space visits of a given map is not uniform but it is peaked around some neighbourhood. Intuitively, one would need to refine the partition in that neighbourhood, to capture fine-grained details which would otherwise be lost. These kind of situations inevitably require a full exploration of the map's phase space prior to any symbolization.

More dramatically, note that the dynamics induced by the symbolization process can in general be very different under different phase space partitions and symbolisations. Concretely, only for so called generating partitions the dynamics after symbolization remain equivalent, although which are the generating partitions is a nontrivial question that lacks a general solution. As a toy example, let us consider the dynamics of a logistic map $f(x) = rx(1 - x)$ in $[0, 1]$, where standard symbolic dynamics uses $p = 2$. For $r = 3.6$ slightly below the first Misiurewicz point, the map is chaotic, but the chaotic attractor is splitted into two disjoint sets. Orbits in this case densely fill the attractor, although they make 'jumps' between each chaotic band in a periodic manner. Therefore, if we

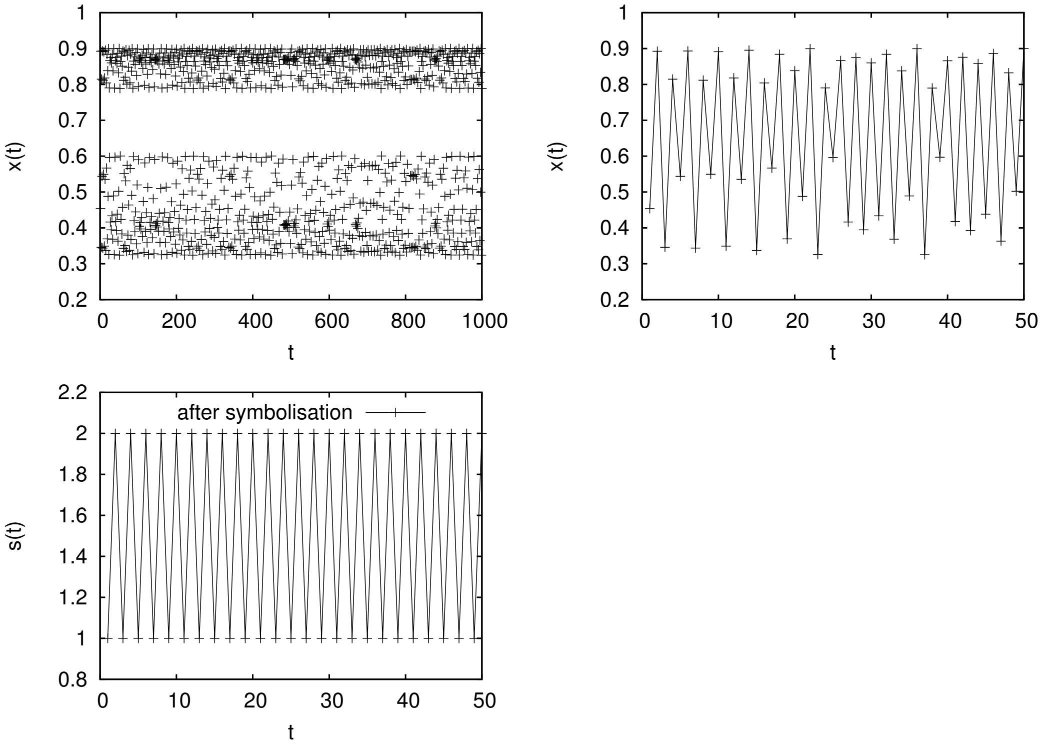

FIG. 1: The first panel is a time series extracted from the logistic map $f(x) = rx(1-x)$ for $r = 3.6$, where the system is chaotic and the attractor is made of two disjoint bands. A zoom of the orbit is presented in the right panel. After symbolisation with $p = 2$ symbols ($s_1 \equiv 1$, $s_2 \equiv 2$) and asymmetric partition $[0,1] = [0,0.7) \cup [0.7,1]$, the induced orbit is periodic of period 2 (bottom panel).

were to partition the phase space in this case as $[0,1] = [0,0.7) \cup [0.7,1]$ (with cells $c_1 = [0,0.7), c_2 = [0.7,1]$) the induced dynamics would become totally periodic, and every trace of the chaotic dynamics would be lost (see figure 1 for an illustration if this toy example). An autocorrelation function or a frequency analysis (power spectrum) -options that do not require a series symbolisation- would also misleadingly conclude that the system is in a periodic state. Interestingly enough, in such a (pathological) toy model, the visibility approach is not misleaded [2].

Note also that finite size effects can play an important role in the symbolisation preprocessing. If the series under study is too short, an excessive number of symbols would inevitably introduce spurious finite size effects, and accordingly, every measure based on the statistics of the symbolised series would be artificially biased. The optimal number of symbols therefore usually depends as well on the size of the series under study.

Finally, it is worth recalling that value of measures computed from symbolised series, such as the well known Shannon entropy, depend on the particular symbolisation, and hence are not invariant under smooth coordinate changes [1] (or under simple changes of experimental units!).
All these issues are usually seen as intrinsic and inevitable drawbacks of the symbolisation method, as this is at least dependent on both p and the specific partition in an *ad hoc* way. Note at this point that the visibility method also induces a symbolisation in the original (multivariate) time series, as a mapping can be straightforwardly defined between the series and the multiplex network's vector integer degree sequence,

$$\{\mathbf{x}(t)\}\underset{\text{HVG}}{\longrightarrow}\{\mathbf{k}(t)\},$$

where $\mathbf{k}(t) \equiv (k^{[1]}(t), k^{[2]}(t), ..., k^{[d]}(t)) \in \mathbb{Z}^d$ and by construction $k^{[\alpha]}(t) > 1$. Within this 'network symbolization', note that the number of symbols p is not a free parameter that needs to be tuned anymore, much on the contrary, the number of symbols emerge naturally by construction and varies from map to map (from series to series). Also, the distribution of symbols is not directly coarse-grained from the distribution of visits to the different regions of the attractor. As a matter of fact, by construction the visibility algorithm makes use of the whole time series

of (continuous) data to generate the degree sequence, and as there is no symbolization prior to this mapping, in principle fine-grained fluctuations are taken into account at all scales.
Furthermore, no *ad hoc* phase space partition needs to be defined within the visibility method. This in principle further removes the second source of ambiguity found for standard symbolization. Moreover, note also that visibility algorithms are invariant under several changes of scale in the original time series [3, 4], hence removing yet another source of ambiguity present in the symbolisation procedure.
Finally, note that whereas the degree sequence can be understood as a time series (global) symbolisation, other structures involving more sophisticated properties of the visibility graphs (degree-degree correlations, spectral properties, etc) can be directly retrieved and hence, at least in principle this method can yield more (or other) information of the dynamical process not described by the symbolised series.

In what follows we compare the performance of the (multiplex) visibility methodology with the results obtained via symbolisation, in the context of diffusively coupled chaotic maps.

II. ORDER PARAMETER AND PHASE DIAGRAM IN DIFFUSIVELY COUPLED CHAOTIC MAPS: A COMPARISON BETWEEN THE MULTIPLEX AND THE TIME SERIES SYMBOLISATION APPROACHES.

We focus on the first system of five diffusively coupled chaotic logistic maps considered in the main text, whose evolution is described by

$$x^{[\alpha]}(t+1) = (1-\epsilon)f[x^{[\alpha]}(t)] + \frac{\epsilon}{2}\bigg(f[x^{[\alpha-1]}(t)] + f[x^{[\alpha+1]}(t)]\bigg). \tag{1}$$

We generate multivariate orbits $\{\mathbf{x}(t)\}_{t=1}^{N}, \mathbf{x} \in \mathbb{R}^5$ of size N and proceed to compute the interlayer averaged mutual information I^{HVG} via the associated visibility multiplex, as defined in the main text. We then compare this proposed order parameter with an analogous measure I^{SYMB} directly performed in the symbolized series. Without loss of generality, for a symbolized series with p integer symbols, let us first define the pairwise mutual information

$$I^{\text{SYMB}}_{\alpha,\beta} = \sum_{s^{[\alpha]}=1}^{p} \sum_{s^{[\beta]}=1}^{p} P(s^{[\alpha]}, s^{[\beta]}) \log \frac{P(s^{[\alpha]}, s^{[\beta]})}{P(s^{[\alpha]})P(s^{[\beta]})} \tag{2}$$

such that

$$I^{\text{SYMB}} = \langle I^{\text{SYMB}}_{\alpha,\beta} \rangle_{\alpha,\beta}.$$

In [5], $p = 2$ and a homogeneous phase space partition was used, but other choices can be made as well. In what follows we compare the performance of I^{HVG} and I^{SYMB} as order parameters that encapsulate the rich dynamical transitions that the system of CMLs undergo as we increase the coupling constant. Concretely, we investigate I^{SYMB} for (i) different number of symbols p, and (ii) different degrees of heterogeneity of the partition. Finally we investigate the effect of shortening the size of the multivariate series under study, N. Such assessment is based in three different criteria, namely (i) capacity of capturing the monotonic increase of synchronisation for weak coupling ($\epsilon < 0.1$, inside the Fully Developped Turbulent (FDT) phase), (ii) accuracy to detect major dynamical transitions (such as the transition from FDT to a randomly selected pattern (PS), or the FDT to Spatio-temporal Intermittency (STI)) through sharp changes in the order parameter, and (iii) accuracy to detect secondary dynamical transitions, such as the onset of multiband chaotic attractors inside the STI phase.
In what follows, we show that the visibility approach is *at least as accurate* as the *optimal* symbolisation (i.e., the optimal selection of symbols and partition). Since this optimal selection is not known a priori, we conclude that the visibility approach is, at least in the case under study, more efficient.

A. Effect of the number of symbols for a homogeneous phase space partition

Let us first fix an homogeneous partition of the phase space $[0,1]^d$ for which cells are hypercubes of dimension d with size $1/p$, where p is the number of symbols considered, and let us explore the effect of varying p on the performance of I^{SYMB}. Note at this point that, if we only consider the 'symbolization' aspect of the visibility algorithm, remind that noisy series have an associated HVG with a mean degree $\langle k \rangle = 4$. Therefore we might consider that, in a first approximation, the HVG method should indeed be comparable to a symbolization with $p \approx 4$. We summarise

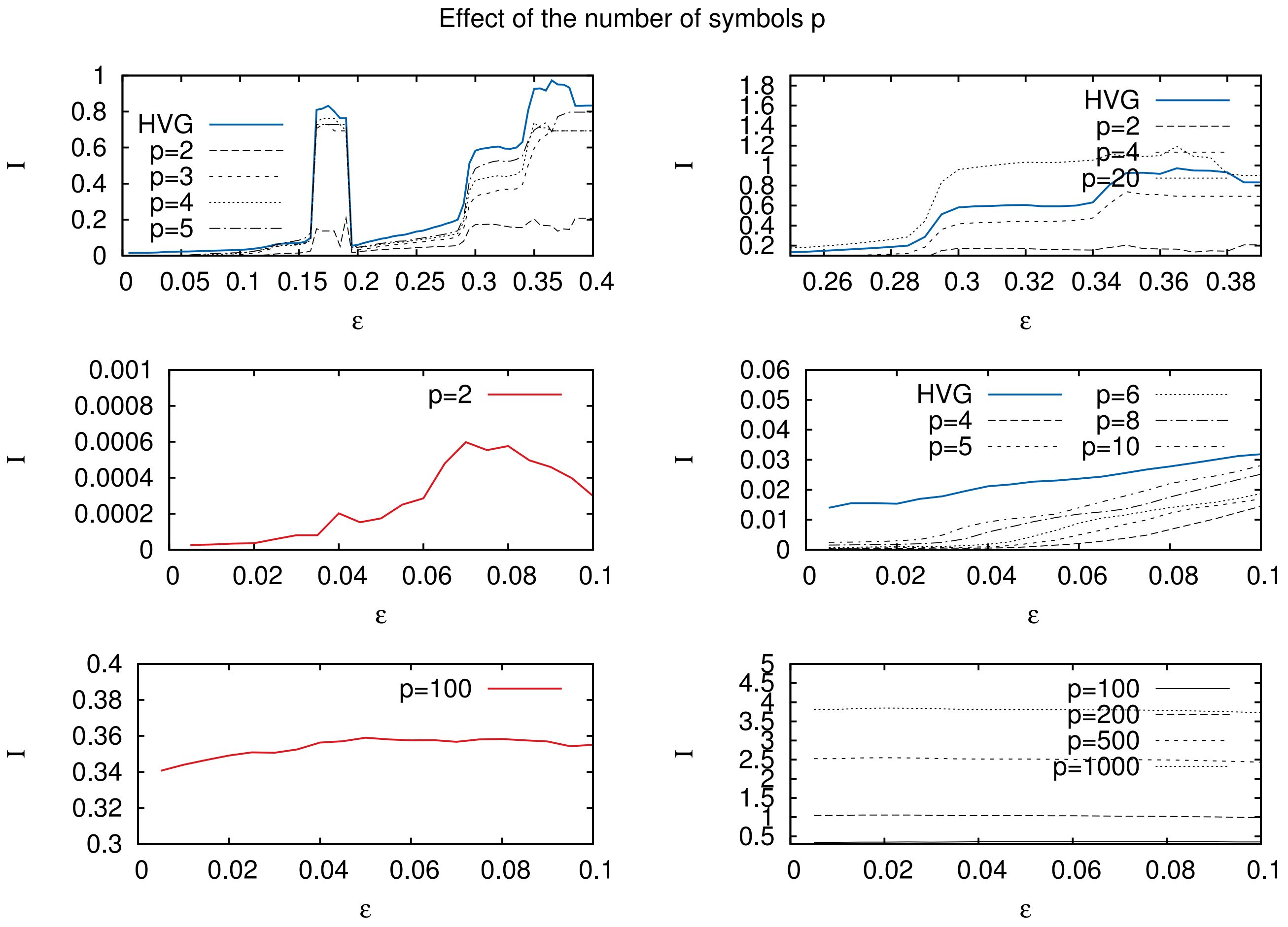

FIG. 2: (color online)*(First row)* Left panel: Averaged mutual information as a function of the coupling intensity ϵ, using the visibility multiplex (blue line for I^{HVG}) and using the symbolizes series with different symbols (yielding I^{SYMB}). Grosso modo, with $p = 5$ both curves are qualitatively similar. Right panel: zoom of the left panel into the STI phase. For $\epsilon \approx 0.34$, the chaotic attractor splits into different bands, and such secondary transition is captured by a sharp change in the visibility multiplex mutual information (blue). Note that in the case of symbolized series, both for small number of symbols ($p = 2$) as well as for $p > 10$, this transition is not captured. *(Second row)* In the left panel, we plot the behavior of I^{SYMB} with $p = 2$ in the weak coupling regime ($\epsilon < 0.1$), where the system displays spatio-temporal chaos (FDT phase) and the mutual information among maps increase monotonically (but weakly) with ϵ. We find that I^{SYMB} is not monotonic in this region. In the right panel we plot the same results for different symbols p, recovering the correct monotonic increasing found with I^{HVG} when the number of symbols is increased. *(Third row)* In the left panel, we plot I^{SYMB} with $p = 100$ symbols again the weakly coupling regime. The monotonic increasing shape is lost again if the number of symbols is too large. This is confirmed in the right panel, concluding that for large number of symbols I^{SYMB} doesn't capture the subtle mutual information increase with ϵ and misleadingly predicts constant mutual information.

our results in figure 2. As expected, qualitatively speaking, the visibility approach yields similar results as the symbolisation method for $p = 4, 5$. For the standard symbolisation (the one used in [5]) with $p = 2$, symbolisation fails to accurately describe several properties such as the increase of synchronisation for weak coupling and the onset of multiband attractors inside STI. Also, for large values $p > 10$, these properties are not captured anymore by the symbolised series.

B. Effect of phase partitioning for a fixed number of symbols

Let us fix now the number of symbols to its standard value $p = 2$, and consider a generic phase partition of the interval $[0, 1] = [0, c] \cup (c, 1]$. Here we explore the performance of I^{SYMB} for different values of $c \in [0, 1]$. We find that

Criterion	HVG	$c = 0.1$	$c = 0.2$	$c = 0.3$	$c = 0.4$	$c = 0.5$	$c = 0.6$	$c = 0.7$	$c = 0.8$	$c = 0.9$
Increase FDT (weak coupling)	YES	NO	YES	YES	YES	NO	NO	NO	NO	NO
Transition to PS	YES	NO	NO	NO	NO	YES	YES	YES	YES	NO
Transition to STI	YES	NO	NO	NO	NO	YES	YES	YES	YES	YES
Onset of multiband chaotic attractor	YES	NO	NO	NO	NO	NO	YES	YES	YES	NO

TABLE I: Capacity of both I^{HVG} and I^{SYMB} (with $p = 2$ symbols and an heterogeneous phase space partition $[0, c) \cup [c, 1]$) to accurately describe several dynamical properties, such as (i) monotonical increase of synchronisation in the weak coupling ($\epsilon < 0.1$) FDT regime, (ii) on and off transition from FDT to PS, (iii) Transition from FDT to STI, (iv) Onset of multiband chaotic attractor inside the STI phase.

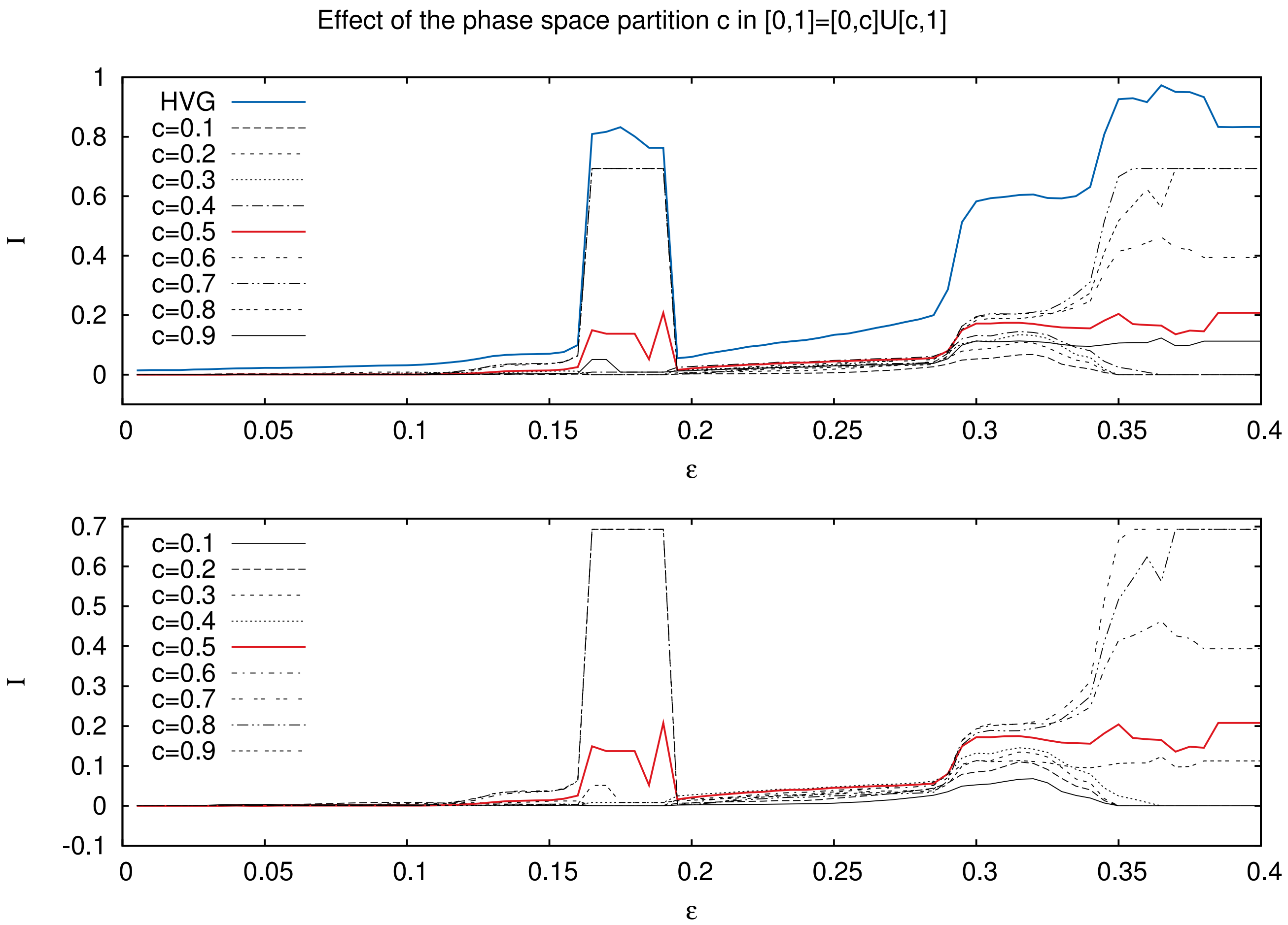

FIG. 3: (color online)I^{HVG} and I^{SYMB} (using the symbolized series with $p = 2$ symbols after different phase space partitioning $[0, c) \cup [c, 1]$) as a function of the coupling intensity ϵ. The symbolised method is only able to accurately retrieve a subset of the main dynamical properties (monotonic increase of I^{SYMB} with the coupling constant in FDT phases, transition between FDT, PS, STI, location of secondary transitions such as onset of multiband chaotic attractors) for particular values c. Interestingly, the usual homogeneous value $c = 0.5$ is shown to be suboptimal, as for this case the symbolization fails to capture the onset of multiband chaotic attractor (around $\epsilon \approx 0.35$), something that a nonstandard partition with $c = 0.6, 0.7, 0.8$ can do.

results dramatically depend on the selection of c and are summarised in table I and in figure 3. We haven't found, for the case $p = 2$, a proper partition that gathers better or equal results than the visibility method. Interestingly, the standard (homogeneous) phase space partition ($c = 0.5$), used in [5], is shown to be suboptimal when compared to other selections such as $c = 0.7 - 0.8$.

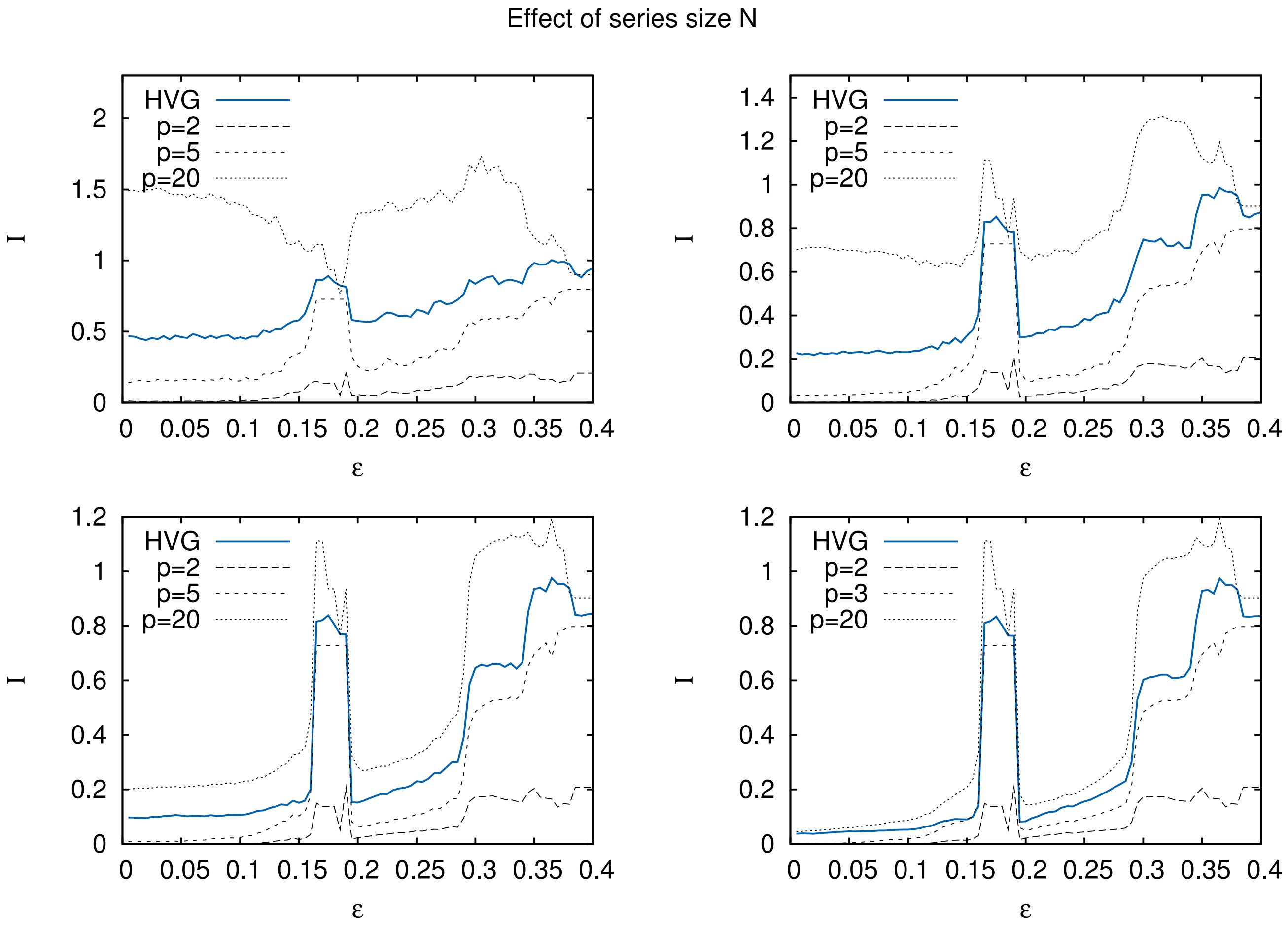

FIG. 4: (color online)Comparison between $I^{\rm HVG}$ and $I^{\rm SYMB}$ as a function of the coupling constant ϵ, for different number of symbols p, for time series of increasing size, from left to right: $N = 2^6$, 2^8, 2^{10} and 2^{12} respectively. Notice that the HVG method is quite robust against shortening, as for sizes of 2^8 we already find the correct profile, including a monotonically increasing function for weak coupling (FDT phase), the location of primary transitions, and the location of onset of multiband chaotic attractor. Similar behaviour is found for $p = 5$, whereas the effect of shortening the size is more acute for larger number of symbols.

C. Effect of time series size for different number of symbols

Finally, we study the effect of shortening the size of the time series. Usually, the shorter the time series, the smaller the upper bound for the number of symbols that can be used before the lack of statistics takes a predominant role and ruins the accuracy of the measurements. Hence the question, which is the 'optimal' number of symbols conditioned to the size of the series under study? Our results are summarised in the panels of figure 4. Roughly speaking, the robustness of both the visibility method and the symbolisation with $p \approx 5$ (the optimal symbolisation according to our previous analysis) against series shortening is similar (the same qualitative results are found in both cases up to short series of size $N = 2^8$ data).

III. EDGE OVERLAP AS AN ALTERNATIVE MEASURE TO DESCRIBE PHASES

We can extend the analysis performed using the pairwise layer averaged mutual information to another multiplex measure. The so called *average edge overlap* $\langle o \rangle$ is defined as:

$$\langle o \rangle = \frac{1}{\mathcal{K}} \sum_{i,j} o_{ij}, \qquad o_{ij} = \frac{1}{M} \sum_{\alpha} a_{ij}^{[\alpha]} \tag{3}$$

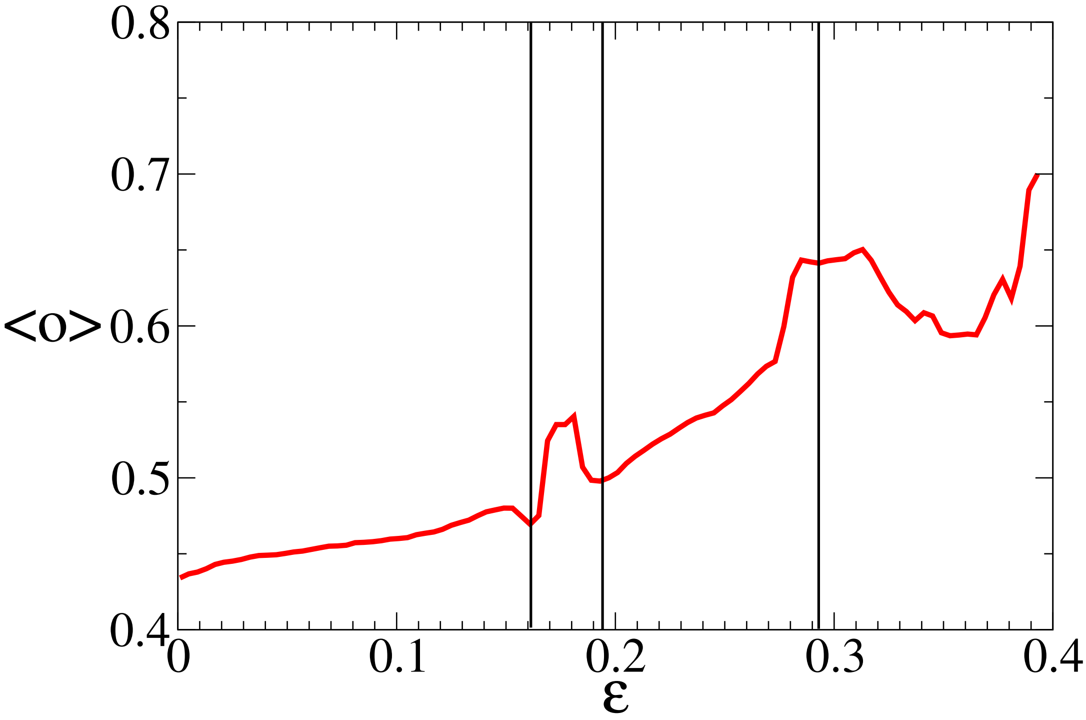

FIG. 5: Average edge overlap of the visibility multiplex as a function of the coupling constant ϵ, computed for the system of five diffusively coupled chaotic maps studied in the main body of the paper.

where o_{ij} is the overlap of the edges between node i and node j at the different layers, and $\mathcal{K}$ is the total number of pairs of nodes connected on at least one of the M layers [6]. Notice that $o_{ij} = 0$ if $a_{ij}^{[\alpha]} = 0 \; \forall \alpha$, while it takes its maximum $o_{ij} = 1$ when nodes i and j are connected at each of the M layers. Consequently, the more similar are the connection patterns of the layers of $\mathcal{M}$ and, in turn, the structure of the corresponding time series, the higher $\langle o \rangle$, with $\langle o \rangle = 1$ if and only if all the layers are identical, i.e. if the original M-dimensional time series can be effectively reduced to a 1-dimensional one.
In figure 5 we plot the average edge overlap for the system of CMLs studied in the main body of the paper, as a function of the coupling constant ϵ. The shape of this purely multiplex measure is qualitatively similar to the pairwise layer averaged mutual information, although subtle dynamical transitions, such as the onset of multiband chaotic attractors, is not clearly seen via this particular metric.

IV. DETECTING THE ONSET OF SYNCHRONISATION IN GLOBALLY COUPLED CHAOTIC MAPS.

To round off the validation part presented in the main body of this paper, we consider here a system of Globally Coupled Maps (GCMs) [7]:

$$x^{[\alpha]}(t+1) = (1-\epsilon) f[x^{[\alpha]}(t)] + \frac{\epsilon}{M} \sum_{\beta=1}^{M} f[x^{[\beta]}(t)] \tag{4}$$

$\forall \alpha = 1, \ldots, M$, where the dynamics of each unit is governed by the logistic map $f(x) = \mu x(1-x), \mu \in [0,4]$. This system can be indeed considered as a mean-field version of CMLs. In particular we consider values of μ for which each map is chaotic, i.e. $\mu > \mu_\infty = 3.56995...$, excluding periodic windows. In those cases, complete synchronization of the GCM is only reached when the system is in the simplest chaotic attractor, where $x^{[\alpha]} = x^{[\beta]} \; \forall \alpha, \beta$, and the dynamics effectively reduces to that of a single logistic map. It can be proved [7] that this is indeed the stable regime for those values of ϵ for which the Lyapunov exponent λ_0 of one isolated logistic map satisfies the inequality

$$\lambda_0 + \log(1-\epsilon) < 0.$$

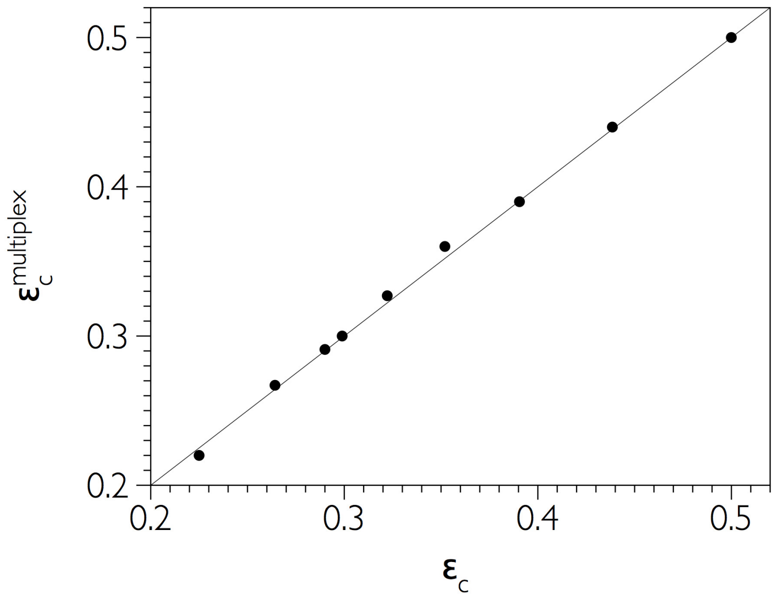

FIG. 6: Scatter plot of the critical value of the coupling strength for the onset of complete chaotic synchronization, as predicted by the multiplex visibility graph $\epsilon_c^{\text{multiplex}}$, versus the theoretical value ϵ_c.

The onset of complete synchronization is therefore reached at a critical value of the coupling strength

$$\epsilon_c = 1 - \exp(\lambda_0)^{-1}.$$

For example at $\mu = 4$, by making use of the the analytic expression $\lambda_0 = \log 2$, we get that the onset of complete synchronization occurs at $\epsilon_c = 1/2$, whereas for other values of μ the value of ϵ_c can be derived from the numerical evaluation of λ_0. The multiplex visibility graph approach is able to predict the position of the onset of complete synchronization. We conjecture that the average mutual information I attains its maximum at the onset of complete synchronization, and accordingly we propose the quantity

$$\epsilon_c^{\text{multiplex}} \equiv \min_{\epsilon}(\text{argmax}(I(\epsilon)))$$

as a measure of ϵ_c. In Fig. 6 we plot $\epsilon_c^{\text{multiplex}}$ versus ϵ_c for a system of 5 globally coupled chaotic maps and for different values of $\mu \in [\mu_\infty, 4]$, finding a remarkable agreement in every case.

V. ANALYSIS OF MULTIVARIATE FINANCIAL SERIES

We have analyzed a large dataset of financial stocks comprising stock evolution of the 35 major american companies from the New York Stock Exchange (NYSE) and Nasdaq in the period 1998-2012, the majority of which belong to the Dow Jones Industrial Average (see table II for a detailed list of all the companies). The NYSE is the largest and most liquid cash equities exchange in the world by market capitalization. The series have very high resolution (one data per minute), yielding $O(2 \cdot 10^6)$ data per company.

Acknowledgments

V.N. and V.L. acknowledge support from the Project LASAGNE, Contract No.318132 (STREP), funded by the European Commission.

[1] H. Kantz and T. Schreiber, Nonlinear Time Series Analysis (Cambridge University Press 2006).

**Acronym**	**Name**	**Comment**
aa	Alcoa Inc.	NYSE
aig	American International Group, Inc.	NYSE
axp	American Express Company	NYSE
ba	The Boeing Company	NYSE
bac	Bank of America Corporation	NYSE
c	Citigroup Inc.	NYSE
cat	Caterpillar Inc.	NYSE
csco	Cisco Systems, Inc.	NYSE
cvx	Chevron Corporation	NYSE
dd	E.I. du Pont de Nemours and Company	NYSE
dis	The Walt Disney Company	NYSE
ge	General Electric Company	NYSE
gm	General Motors Company	NYSE
hd	The Home Depot, Inc.	NYSE
hon	Honeywell International Inc.	NYSE
hpq	Hewlett-Packard Company	NYSE
ibm	International Business Machines Corporation	NYSE
intc	Intel Corporation	NasdaqGS
jnj	Johnson & Johnson	NYSE
jpm	JPMorgan Chase & Co.	NYSE
ko	The Coca-Cola Company	NYSE
mcd	McDonald's Corp.	NYSE
mmm	3M Company	NYSE
mo	Altria Group Inc.	NYSE
mrk	Merck & Co. Inc.	NYSE
msft	Microsoft Corporation	NasdaqGS
pfe	Pfizer Inc.	NYSE
pg	The Procter & Gamble Company	NYSE
t	AT&T, Inc.	NYSE
trv	The Travelers Companies, Inc.	NYSE
unh	UnitedHealth Group Incorporated	NYSE
utx	United Technologies Corporation	NYSE
vz	Verizon Communications Inc.	NYSE
wmt	Wal-Mart Stores Inc.	NYSE
xom	Exxon Mobil Corporation	NYSE

TABLE II: List of companies

[2] A. Nuñez, L. Lacasa, B. Luque, *Int. J. Bif. Chaos* **22**, 7 (2012).
[3] L. Lacasa, B. Luque, F. Ballesteros, J. Luque, J.C. Nuno, *Proc. Natl. Acad. Sci. USA* **105**, 13 (2008).
[4] B. Luque, L. Lacasa, J. Luque, F.J. Ballesteros, *Phys. Rev. E* **80**, 046103 (2009).
[5] K. Kaneko, *Physica D* **34**, 1–41 (1989).
[6] F. Battiston, V. Nicosia, V. Latora, *Phys. Rev. E* **89**, 032804 (2014).
[7] K. Kaneko, *Physica D* **41**, 137-172 (1990).
